# Conservation of the Amyloid Interactome Across Diverse Fibrillar Structures

**DOI:** 10.1038/s41598-019-40483-z

**Published:** 2019-03-07

**Authors:** Dennis Wilkens Juhl, Michael Wulff Risør, Carsten Scavenius, Casper Bøjer Rasmussen, Daniel Otzen, Niels Chr. Nielsen, Jan J. Enghild

**Affiliations:** 1 Interdisciplinary Nanoscience Center (iNANO), Aarhus University, DK-8000 Aarhus, Denmark; 2 Department of Molecular Biology and Genetics, Aarhus University, Aarhus, Denmark; 3 Department of Chemistry, Aarhus University, Aarhus, Denmark

## Abstract

Several human proteins cause disease by misfolding and aggregating into amyloid fibril deposits affecting the surrounding tissues. Multiple other proteins co-associate with the diseased deposits but little is known about how this association is influenced by the nature of the amyloid aggregate and the properties of the amyloid-forming protein. In this study, we investigated the co-aggregation of plasma and cerebrospinal proteins in the presence of pre-formed amyloid fibrils. We evaluated the fibril-associated proteome across multiple amyloid fibril types that differ in their amino acid sequences, ultrastructural morphologies, and recognition by amyloid-binding dyes. The fibril types included aggregates formed by Amyloid β, α-synuclein, and FAS4 that are associated with pathological disorders, and aggregates formed by the glucagon and C-36 peptides, currently not linked to any human disease. Our results highlighted a highly similar response to the amyloid fold within the body fluid of interest. Fibrils with diverse primary sequences and ultrastructural morphologies only differed slightly in the composition of the co-aggregated proteins but were clearly distinct from less fibrillar and amorphous aggregates. The type of body fluid greatly affected the resulting amyloid interactome, underlining the role of the *in vivo* environment. We conclude that protein fibrils lead to a specific response in protein co-aggregation and discuss the effects hereof in the context of amyloid deposition.

## Introduction

The amyloid structural arrangement is a generic fold^1,2^ that most peptides and many proteins can adopt under a wide range of *in vitro* conditions. Generally, these conditions are very destabilizing (*e*.*g*. extreme pH, elevated temperature, and presence of organic solvents), ensuring that the vast majority of proteins remains stably folded *in vivo*^3^. However, a small selection of proteins can fibrillate under physiological conditions and therefore deposit as amyloid structures over long periods of time. Protein misfolding and aggregation into amyloid deposits is associated with more than 25 human diseases^4^, and constitutes a hallmark of the well-known neurodegenerative disorders like Alzheimer’s^5^ (AD) and Parkinson’s disease^6^ (PD). Immunostaining assays^7^ and tandem mass-spectrometry (MS/MS) analyses^8^ have revealed how each type of deposit is linked to the misfolding of one particular protein that constitutes the majority of the aggregate mass^4^. The formation of amyloid deposits occurs in several different tissue types and the exact localization and spread depends on the identity of the misfolded protein and the severity of the disease^4^. Examples include extracellular β-amyloid peptide (Aβ) deposits in the brain of AD patients^9^, intracellular Lewy body deposition of α-synuclein (αSN) in the brain of PD patients^10^, transforming growth factor β-induced protein (TGFBIp) accumulation in the cornea^11^, and plaque formation of the immunoglobulin light chain protein in hearts, kidneys, and livers of patients suffering from amyloid light-chain (AL) amyloidosis^12^.

The native fold of amyloid-forming proteins vary greatly, but in the amyloid deposits, the main component shares a similar fibrillar appearance with an underlying highly ordered and repetitive β-sheet arrangement independent of the disease^13^. This structural property provides a characteristic cross-β X-ray diffraction pattern and apple-green birefringence under polarized light with Congo-red staining. Detailed structural studies of the fibrillar deposits with EM, atomic force microscopy, X-ray crystallography, and NMR have revealed distinct variations in the exact molecular arrangement of fibrils, which involves the fibrillar morphology, the stacking of the cross-β spine, and the atomic level details of the repetitive protein unit^14,15^. These variations can be the result of the protein’s primary sequence but to a large extent also the conditions under which fibril formation took place^16,17^. For both Aβ and αSN, atomic-level details of several molecular arrangements now exist^18–21^. While the functional consequences of the exact molecular arrangement remain elusive, several studies suggests a link between disease pathology and fibril morphology^22–24^.

Besides the main protein component, amyloid deposits consist of proteoglycans, metal ions, lipids, and other co-aggregated proteins^9^. Proteomic analyses of AD plaques^25,26^, AL deposits^27^, amyloid deposits in Lattice Corneal Dystrophy^28^, and Transthyretin deposits in Familial amyloidotic polyneuropathy disease^29^ present similar lists of co-aggregated proteins that include components of the complement system, growths factors, proteases and protease inhibitors, coagulation factors, and various apolipoproteins. The similarities could indicate a general response of proteins to the formation of fibrillar assemblies but differences between the study design, sample preparation, and the lack of molecular level detail of the fibrils preclude further conclusions. A better understanding of the protein-coating response to the amyloid surface and the influence of the fibril morphology on such a response is warranted in order to elucidate both the importance of co-aggregated proteins and the significance of morphological differences of the underlying amyloid fibril structures.

In this work, we have applied quantitative protein MS and SDS-PAGE analyses to study the composition of proteins that co-aggregate with a panel of preformed fibrils introduced into biological fluids. We compared the amyloid-interacting proteins (fibril interactome) of five different fibrillating systems to each other and to the interactome of an amorphous aggregate to verify fibril specificity. We characterized and validated the amyloid fold of our aggregate structures by electron microscopy and FTIR spectroscopy and employed organic tracers to highlight molecular-level surface property differences between the various fibrils.

The selected amyloid systems and protein aggregates were all prepared from human-origin peptides with published protocols for amyloid formation. Our panel included: (i) Two structurally distinct fibrils of the Aβ(1–40) peptide associated with AD, (ii) fibrils of αSN^10^ associated with Parkinson’s disease, (iii) fibrils of the peptide hormone glucagon used as a model system in biophysical studies of fibrillation^30^, (iv) fibrils of the 36 residue C-terminal fragment of α_1_-antitrypsin (C-36) found in atherosclerotic plaques^31^, and (v) aggregates of a mutated version of the FAS1-4 domain of TGFBIp, one of the major components of the human cornea^32^. The specific A546D mutation (FAS4 AD) is a particularly aggregation-aggressive variant^33,34^ and has been correlated with LCD deposits^35^. Additionally, we prepared trichloroacetic acid (TCA)-induced aggregates of the globular protein chymotrypsinogen^36^ that served as an amorphous aggregate control.

## Results

### Fibrillation and morphological characterization of the amyloid species

**Figure 1:**
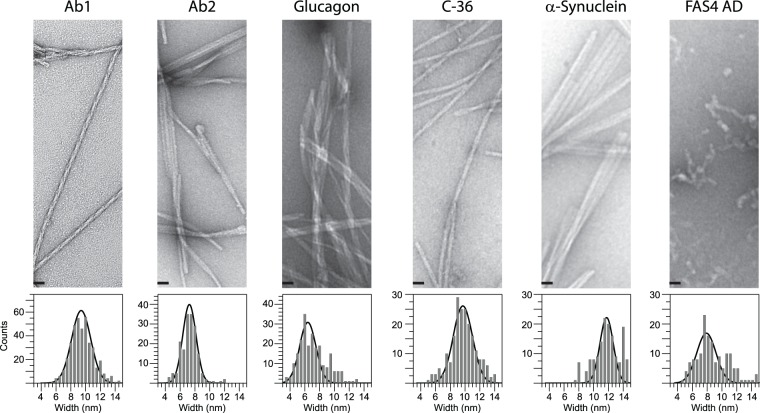
Representative TEM images introducing the morphologies of Ab1, Ab2, Glucagon, C-36 and αSN fibrils together with the FAS4 AD aggregates (*Top*). Scale bars represent 25 nm. The average fibril width was determined to 9.4 ± 1.4 nm (Ab1), 7.3 ± 0.9 nm (Ab2), 6.4 ± 1.1 nm (Glucagon), 9.7 ± 1.3 nm (C-36), 11.8 ± 0.9 nm (αSN), 7.8 ± 1.3 nm (FAS4 AD) by a standard distribution function covering bins of 0.5 nm (*Bottom*). Entire images are presented in SI.

The human amyloid proteins (Aβ, αSN, glucagon, C-36, and FAS4 AD) were reproducibly fibrillated to yield characteristic morphologies as evaluated by EM (Fig. 1). We generated two distinct morphologies of Aβ using either quiescent (Ab1) or agitating (Ab2) conditions followed by several rounds of seeding^37^. The Ab1 morphology appeared as straight single fibrils with a short-pitch twist along the fibril axis while the Ab2 fibrils formed twisted ribbons of three to four protofibrils. Measurements of individual protofibril width were counted in bins of 0.5 nm and modelled by a normal distribution centred on 9.4 ± 1.4 nm for Ab1 and 7.3 ± 0.9 nm for Ab2 (Fig. 1, bottom). For the αSN protein we obtained polymorphic fibril structures with a predominant ribbon arrangement and individual fibril widths of 11.8 ± 0.9 nm. Fibrils of the glucagon peptide formed larger bundles of laterally associated straight fibrils with an individual width of 6.4 ± 1.1 nm. The C-36 fibrils averaged 9.7 ± 1.3 nm in width and formed twisted ribbons. In our hands, the FAS4 AD protein formed “worm-like” aggregate structures with limited fibril appearance and an average width of 7.8 ± 1.3 nm.

By FTIR we confirmed the characteristic amide C=O stretch frequency of the β-sheet arrangement of the amyloid fold^38^ with the amide-I band peaks positioned around 1625 cm^−1^ for Ab1, Ab2, aSN, glucagon, and C-36 fibrils. The FAS4 AD protein aggregates also displayed a similar β-sheet peak but had a higher signal in the non-amyloid region from 1640 to 1680 cm^−1^ (Figure S7). In comparison, the TCA-induced protein aggregate of chymotrypsinogen showed a broad amide-I band from 1625 to 1660 cm^−1^ that similar to other amorphous aggregates^39^ indicates a mix of conformational states and lack of long-range order.

Before applying the samples to the interaction studies, the protein aggregate concentrations were determined by SDS-PAGE and HPLC analysis of denatured aliquots (data not shown). Experimentally, the aggregate systems were studied and compared based on equal amounts of fibril mass.

### Differential ligand recognition reflects differences at the atomic level

**Figure 2:**
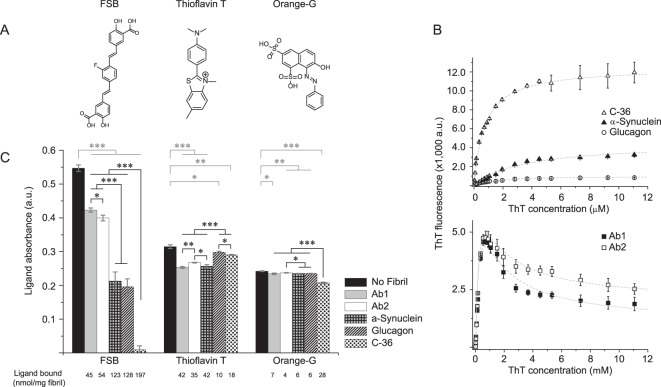
Fibril structure differentiation defined by ligand interaction. (**A**) Molecular structures of the three amyloid tracers FSB, ThT and OG. (**B**) ThT fluorescence intensity (Ex 450 nm, Em 480 nm) as a function of ThT concentration added to 7.5 μg of fibril material. Signal intensities follow a single-site binding model for C-36 (k_d_ 0.63 ± 0.04 μM, I_max_ 12,500 rfu), αSN (k_d_ 3.5 ± 0.1 μM, I_max_ 4,500 rfu) and Glucagon fibrils (k_d_ 1.45 ± 0.08 μM, I_max_ 1,000 rfu) and a two-site binding model for Ab1 (k_d1_ 0.40 ± 0.01 μM, I_max1_ 23,000 rfu, k_d2_ 0.82 ± 0.02 μM, I_max2_ −22,000 rfu) and Ab2 (k_d1_ 0.4 ± 0.01 μM, I_max1_ 23,000 rfu, k_d2_ 0.8 μM ± 0.02, I_max2_ −21,250 rfu). (**C**) Residual ligand absorbance in supernatants of 2 nmol FSB, ThT, and Orange-G after incubation with 10 μg of fibril material. The concentrations were determined by UV/VIS absorbance (Absorbance max: ThT 412 nm, FSB 350 nm, orange-G 480 nm) and significant differences are indicated as *p < 0.05; **p < 0.005 and ***p < 0.001. The data represents three technical replicates with error bars showing the standard deviations.

Thioflavin T fluorescence emission intensity increases significantly when the compound binds to fibrils^40^ but the level of increase depends on the fibril morphology^16,23,33,41^. We explored this property to visualize structural differences at the surface of the fibrils systems included here (Fig. 2B). For glucagon, αSN, and C-36 fibrils, ThT fluorescence signals followed a simple one-site binding model upon increasing ThT concentrations. The extracted dissociation constants (0.63 μM ± 0.04 for C-36, 1.45 μM ± 0.08 for glucagon, and 3.5 μM ± 0.1 for αSN fibrils) reveal slightly different affinities of ThT towards the fluorescence-inducing binding sites of the fibril systems. Additionally, we observed major differences in the maximum ThT fluorescence intensities among the fibril systems with 12,500 relative fluorescence units (rfu) for C-36, 4,000 rfu for αSN, and 1,000 rfu for glucagon.

For the two Aβ fibril morphologies, ThT fluorescence displayed a different concentration dependence compared to the other fibril systems. The fluorescence signal increased up to 1 µM ThT but then gradually decreased. We accounted for the second change in fluorescence intensity by fitting the concentration dependencies to a two-site binding model (Fig. 2B bottom). The dissociation constants were similar for Ab1 and Ab2 (Kd_1_ = 0.40 ± 0.01 μM, Kd_2_ = 0.80 ± 0.02 μM) and only the extent of signal quenching at high ThT concentrations was notably different for the two Aβ fibril systems. The bimodal appearance of the ThT binding curve may be explained by a photochemical quenching effect upon secondary ThT binding or by modification of the binding mode in the high affinity site at high ThT to peptide ratios. Of note, we did not detect any fluorescence when ThT was mixed with FAS4 AD aggregates. As a result hereof and of their worm-like appearance on the EM images, we considered the FAS4 AD aggregates to be non-conventional fibril-like aggregates despite their amyloid-like FTIR signal. As expected, the chymotrypsinogen amorphous aggregates did not result in any ThT signal.

Hydrophobic patches may be exposed to the surface as a consequence of protein fold collapse into aggregate structures and we used titrations of the fluorescent 8-anilinonaphthalene sulfonate (ANS) dye to probe this property for our aggregate systems^42,43^. Our results indicated that only αSN gave significant fluorescence enhancements with ANS (Figure S8). The protein aggregates FAS4 AD and chymotrypsinogen returned intermediate signal intensities while the Ab1, Ab2, C-36, and glucagon fibrils gave little or no signal at all.

To further discriminate between the amyloid surfaces of the selected panel of fibrils, we employed two other amyloidogenic dyes, FSB^44^ and Orange-G^45^, in conjugation with ThT and looked at the total binding capacity for the individual fibrils (Fig. 2A). After co-incubation of 2 nmol dye (20 µM) with 10 μg fibril-material (Fig. 2C), the fibrils were centrifuged into a pellet and the dye concentration in the supernatant determined by UV-VIS absorbance. The FSB dye displayed both the highest binding capacity and the biggest difference between fibril systems. For the two Aβ fibril systems, similar binding capacities of FSB were observed (45 and 54 nmol/mg fibril, respectively), while the capacity was much higher for both αSN and glucagon fibrils (123 and 128 nmol/mg fibril, respectively). The highest FSB binding capacity was observed for the C-36 fibrils which bound 197 nmol FSB per mg of fibril material.

All fibril systems displayed low binding capacities for the Orange-G dye (4, 6, 6, 7 and 28 nmol Orange-G per mg of Ab2, glucagon, αSN, Ab1, and C-36 fibrils, respectively). The low binding of Orange-G is not surprising as its dissociation constant to fibrils of a short Aβ fragment is in the higher micromolar range (~50 μM)^46^.

In contrast to what one might expect, we found no correlation between the total ThT binding capacity and the maximum fluorescence signal observed in the titration experiment for the fibril systems. The Ab1, Ab2, and αSN fibrils bound 42, 35, and 42 nmol of ThT per mg of fibril material, respectively, and had relatively similar ThT fluorescence levels (2–5,000 RFU). However, glucagon and C-36 fibrils bound only 18 and 10 nmol/mg fibril material, respectively, but C-36 had the highest ThT fluorescence level of all the fibrils tested (12,500 RFU). The lack of correlation between the number of ThT molecules bound and the observed ThT fluorescence for the individual fibril systems demonstrates ThT’s ability to report on the system-specific character of the fibrillar surface and not just the binding site accessibility.

Collectively, the organic dyes confirmed that despite a similar secondary structure, the selected amyloid fibrils contain substantially different surface properties. These differences might originate from diverse primary sequences, the specific monomer packing, and the overall morphological variations as observed by EM.

### Identification of the amyloid interactome in plasma and CSF

**Figure 3:**
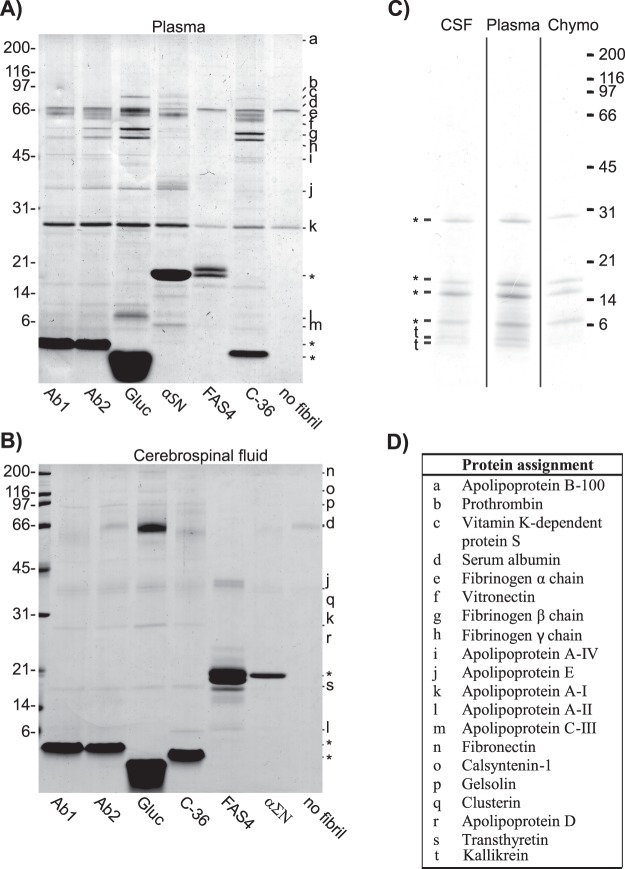
Composition of plasma and CSF proteins interacting with each fibril system. (**A**) SDS-PAGE analysis of fibril material (10 μg) incubated with 500 μg plasma proteins. (**B**) SDS-PAGE analysis of fibril material (10 μg) incubated 40 μg CSF proteins. (**C**) SDS-PAGE analysis of precipitated chymotrypsinogen (10 μg) with 500 μg plasma proteins and 40 μg CSF proteins, respectively. (**D**) Identification of the indicated protein bands was performed by MS/MS. The position of the fibril peptides has been marked by asterisk, *.

By SDS-PAGE and mass spectrometry analyses, we compared the composition of plasma proteins which associated with the different fibrils and aggregates after co-incubation at a 50:1 w/w plasma protein:fibril ratio (Fig. 3A). The composition of proteins was remarkably similar in protein type and quantity among the fibril systems. Only the glucagon fibrils showed a slightly higher binding for some proteins.

By LC-MS/MS we identified the co-aggregated proteins contributing to the major bands in all lanes. These proteins were apolipoprotein (Apo) A-I, ApoA-II, ApoA-IV, ApoB-100, ApoE, serum albumin, and fibrinogen α−, β−, and γ−chain. We identified prothrombin exclusively in the lanes containing glucagon, αSN, and C-36 fibrils, whereas vitamin K-dependent protein S and ApoC-III were found exclusively with αSN. For the non-fibrillar FAS4 AD aggregates, only serum albumin and ApoA-I could be identified.

We performed a similar analysis in CSF with a 4:1 w/w CSF protein:fibril ratio. The low ratio compared to plasma was caused by the much lower total protein concentration in CSF compared to plasma. The protein bands were less intense compared to plasma, but we observed a conserved pattern across the C-36, glucagon, Ab1, and Ab2 fibril lanes (Fig. 3B). Based on LC-MS/MS, we identified the major co-aggregated proteins as fibronectin, calsyntenin-1, gelsolin, serum albumin, clusterin, ApoA-I, ApoA-II, ApoD, ApoE, and transthyretin. For the αSN fibrils, we only observed a few weak bands which we identified as fibronectin, serum albumin, ApoE, and clusterin. Similar to the analysis in Plasma, the FAS4 AD aggregates only resulted in weak bands, here identified as ApoA-II, ApoE, and clusterin.

The absence of any noticeable co-aggregation with the FAS4 AD aggregates suggests that their non-fibrillar architecture presents a different surface to the surrounding fluids. Of note, FAS4 does have the capacity to form bona fide amyloid fibril deposits *in vivo*^47^ and *in vitro*^33,34^ but did not do so in our *in vitro* setting.

To further evaluate if the observed interactions are specific for fibril architecture and not a consequence of non-specific binding to aggregated proteins, we also incubated plasma and CSF with TCA-induced amorphous aggregates of chymotrypsinogen. Besides several bands from chymotrypsinogen itself, SDS-PAGE identified only two weak low molecular bands from Kallikrein in the aggregates after incubation (Fig. 3C). The low association of proteins to the amorphous chymotrypsinogen aggregates supports the role of the amyloid fold in causing fibril-specific protein association rather than non-specific co-aggregation.

We also tested for the presence of protein aggregates in the absence of fibril material, which for plasma resulted in weak bands from serum albumin and ApoA-I, and for CSF only resulted in a weak band from serum albumin, most likely caused by residual plasma or CSF left during sample preparation.

### Quantitative conservation of the protein composition in the amyloid interactome

We used a quantitative mass spectrometry approach to characterize the amount and composition of proteins co-aggregated with each fibril sample. A total of 74 plasma proteins and 84 CSF proteins were quantified across the fibril systems. For each sample, we normalized the individual peptide’s ion intensity to the total intensity of all peptides. The proteins with a major contribution to the interactome of any of the fibrils are listed in Tables 1 and 2 (>1% in plasma, >0.5% in CSF) together with the total ion intensity for each fibril system. We observed a highly-conserved interactome composition across the fibril systems (Ab1, Ab2, glucagon, αSN and C-36) with minor differences in the relative contribution from individual proteins and protein types. This also applied to the low amount of CSF proteins bound to αSN fibrils which suggests that the nature of the protein-fibril co-aggregation is similar for all the fibril systems. We also determined the relative protein composition of plasma and CSF and showed that this composition was different compared to the fibril interactomes. Overall, we consider the co-aggregation of proteins to be specific for fibrillar structures and not merely a reflection of the original body fluid composition. The specificity of the interaction is underlined by the lack of quantifiable protein IDs for the amorphous chymotrypsinogen aggregates.

**Table 1:** Relative composition of plasma proteins bound to each fibril system.

		Plasma	Ab1	Ab2	Glucagon	C-36	αSN	FAS4-AD
	Total protein ion count	1.4 ·10^6^	1.1 ·10^5^	6.8 ·10^4^	1.8 ·10^5^	1.8 ·10^5^	5.3 ·10^4^	9.7 ·10^3^
	*Apolipoproteins (% of total protein)*	2.7±0.2	22.5 ± 1.9	29.0 ± 10.7	24.4 ± 2.5	29.2 ± 3.1	45.1 ± 12.8	35.2 ± 11.0
P02647	Apolipoprotein A-I^#^	1.9 ± 0.1	8.8 ± 0.9	14.9 ± 7.0	11.7 ± 1.4	13.5 ± 0.5	14.7 ± 3.0	15.7 ± 4.6
P02652	Apolipoprotein A-II^#^	0.2 ± 0.0	1.1 ± 0.2	1.2 ± 0.2	6.2 ± 0.6	1.9 ± 0.5	0.9 ± 0.3	1.8 ± 0.2
P06727	Apolipoprotein A-IV^#^	0.2 ± 0.0	2.7 ± 0.1	3.5 ± 0.5	1.9 ± 0.2	2.4 ± 0.4	3.8 ± 0.6	1.6 ± 0.2
P04114	Apolipoprotein B-100	0.2 ± 0.0	0.6 ± 0.1	0.5 ± 0.2	0.4 ± 0.0	0.1 ± 0.0	0.2 ± 0.1	*
P02654	Apolipoprotein C-I	0.0 ± 0.0	*	*	*	0.6 ± 0.4	5.5 ± 2.1	1.8 ± 0.1
P02656	Apolipoprotein C-III^#^	0.1 ± 0.1	0.9 ± 0.1	*	0.3 ± 0.0	4.9 ± 0.3	11.7 ± 3.0	*
P05090	Apolipoprotein D	0.0 ± 0.0	0.1 ± 0.0	0.3 ± 0.0	0.3 ± 0.1	0.9 ± 0.1	*	1.1 ± 0.1
P02649	Apolipoprotein E^#^	0.1 ± 0.0	7.4 ± 0.3	6.4 ± 1.5	3.0 ± 0.1	1.3 ± 0.1	6.8 ± 2.9	5.4 ± 1.6
P10909	Clusterin	0.0 ± 0.0	1.1 ± 0.1	2.2 ± 1.2	0.5 ± 0.1	3.5 ± 0.9	1.6 ± 0.9	7.9 ± 4.2
	***Immunoglobulins (% of total protein)***	**22.9** ± **0.6**	**4.9** ± **0.4**	**5.2** ± **0.9**	**5.5** ± **0.5**	**4.8** ± **0.7**	**4.5** ± **2.1**	**23.1** ± **7.8**
P01876	Ig α-1 chain C region	3.2 ± 0.1	1.3 ± 0.1	1.3 ± 0.3	1.7 ± 0.0	1.4 ± 0.4	0.9 ± 0.3	3.9 ± 1.6
P01857	Ig γ-1 chain C region	6.7 ± 0.2	1.1 ± 0.1	1.2 ± 0.1	1.2 ± 0.2	1.9 ± 0.2	1.9 ± 0.9	9.5 ± 2.6
P01859	Ig γ -2 chain C region	6.0 ± 0.1	0.9 ± 0.1	0.9 ± 0.1	0.9 ± 0.1	1.2 ± 0.2	1.2 ± 0.8	7.2 ± ± 2.0
P01860	Ig γ -3 chain C region	6.7 ± 0.2	1.0 ± 0.1	1.1 ± 0.1	1.0 ± 0.1	*	*	—
P01871	Ig μ chain C region	0.3 ± 0.0	0.6 ± 0.0	0.7 ± 0.2	0.8 ± 0.1	0.4 ± 0.1	0.5 ± 0.2	2.6 ± 1.5
	***Blood coagulation (% of total protein)***	**4.5** ± **0.1**	**41.1** ± **2.4**	**30.1** ± **3.7**	**30.5** ± **2.1**	**35.0** ± **4.2**	**30.4** ± **7.0**	**6.6** ± **1.8**
P01009	α_1_-antitrypsin	1.6 ± 0.1	0.4 ± 0.0	0.4±0.0	0.6 ± 0.0	*	*	1.4 ± 0.5
P00740	Coagulation factor IX	*	0.2 ± 0.0	0.3 ± 0.1	0.1 ± 0.0	0.2 ± 0.0	0.3 ± 0.1	*
P00742	Coagulation factor X	*	0.4 ± 0.0	0.5 ± 0.1	0.6 ± 0.0	0.2 ± 0.1	0.9 ± 0.5	*
P02671	Fibrinogen α chain^#^	0.9 ± 0.0	14.8 ± 0.8	10.9 ± 1.9	11.0 ± 0.3	9.4 ± 0.5	9.8 ± 2.4	1.9 ± 0.6
P02675	Fibrinogen β chain^#^	1.3 ± 0.1	11.8 ± 1.1	7.5 ± 0.9	8.9 ± 1.1	9.1 ± 0.8	7.7 ± 2.3	1.8 ± 0.2
P02679	Fibrinogen γ chain^#^	0.6 ± 0.0	9.8 ± 0.3	7.0 ± 0.3	6.7 ± 0.3	11.7 ± 2.0	7.7 ± 1.3	1.5 ± 0.4
P02751	Fibronectin	0.0 ± 0.0	0.1	±	0.0	0.2 ± 0.0	1.0 ± 0.0	0.3 ± 0.1
P04196	Histidine-rich glycoprotein	0.0 ± 0.0	2.5 ± 0.1	1.8 ± 0.1	0.4 ± 0.0	—	—	—
P00734	Prothrombin^#^	0.1 ± 0.0	*	*	0.6 ± 0.1	3.5 ± 0.5	1.4 ± 0.4	—
P07225	Vitamin K-dependent protein S^#^	0.0 ± 0.0	1.1 ± 0.0	1.3 ± 0.1	0.8 ± 0.1	0.6 ± 0.1	2.5 ± 0.1	*
	***Complement system (% of total protein)***	**0.5** ± **0.0**	**8.7** ± **0.4**	**14.4** ± **1.1**	**6.6** ± **0.5**	**7.2** ± **0.6**	**8.7** ± **2.8**	**2.7** ± **0.9**
P04003	C4b-binding protein α chain	0.1 ± 0.0	6.1 ± 0.2	9.9 ± 0.5	3.0 ± 0.1	5.5 ± 0.3	7.4 ± 2.5	2.7 ± 0.9
P20851	C4b-binding protein β chain	0.0 ± 0.0	0.9 ± 0.1	1.3 ± 0.3	0.5 ± 0.1	0.7 ± 0.1	1.3 ± 0.4	*
P00736	Complement C1r subcomponent	*	0.2 ± 0.0	0.4 ± 0.1	0.1 ± 0.0	0.3 ± 0.1	*	—
P09871	Complement C1s subcomponent	0.0 ± 0.0	0.3 ± 0.0	0.7 ± 0.1	0.2 ± 0.0	0.4 ± 0.1	*	—
P01024	Complement C3	0.4 ± 0.0	0.4 ± 0.0	0.6 ± 0.1	2.3 ± 0.2	0.2 ± 0.0	*	*
P0C0L4	Complement C4-A	*	0.9 ± 0.0	1.6 ± 0.1	0.5 ± 0.1	*	*	*
	***Other proteins (% of total protein)***	**53.5** ± **2.5**	**21.2** ± **2.0**	**17.6** ± **2.7**	**27.2** ± **3.3**	**12.9** ± **1.0**	**17.8** ± **5.1**	**24.7** ± **3.5**
Q14520	Hyaluronan-binding protein 2	*	1.4 ± 0.0	1.6 ± 0.3	0.5 ± 0.0	0.4 ± 0.1	1.4 ± 0.8	*
P19823	Inter-α-trypsin inhibitor heavy chain H2	0.2 ± 0.0	0.1 ± 0.0	0.1 ± 0.0	2.7 ± 0.2	0.3 ± 0.1	*	—
P02768	Serum albumin	53.1 ± 2.5	2.5 ± 0.0	3.5 ± 0.2	19.9 ± 3.0	7.3 ± 0.4	7.2 ± 2.1	23.1 ± 3.2
P04004	Vitronectin^#^	0.2 ± 0.0	17.1 ± 1.9	12.1 ± 2.1	3.7 ± 0.1	4.9 ± 0.5	9.3 ± 2.2	1.6 ± 0.4

Proteins constituting > 1% of the total composition, for at least one fibril system are displayed. Respective fibril peptides have been removed from the quantification. The data represents nine technical replicas with standard deviations.

*Identified, but not quantified. —Not identified in any of the samples. ^#^Significant band in gel analysis.

**Table 2:** Relative composition of CSF proteins bound to each fibril system.

		CSF	Ab1	Ab2	Glucagon	C-36	αSN	FAS4-AD
	Total protein ion count	1.2 ·10^6^	4.4 ·10^4^	2.6 ·10^4^	2.6 ·10^5^	6.5 ·10^4^	5.0 ·10^3^	3.5 ·10^3^
	*Apolipoproteins (% of total protein)*	1.7 ± 0.1	26.3 ± 2.7	21.9 ± 3.1	11.2 ± 3.4	30.2 ± 8.0	30.8 ± 7.5	36.2 ± 12.8
P02647	Apolipoprotein A-I^#^	0.4 ± 0.1	3.9 ± 0.4	2.9 ± 0.6	2.6 ± 0.8	5.0 ± 1.6	2.7 ± 1.0	*
P02652	Apolipoprotein A-II^#^	0.0 ± 0.0	1.0 ± 0.3	*	0.6 ± 0.2	0.9 ± 0.1	—	—
P06727	Apolipoprotein A-IV	0.0 ± 0.0	1.2 ± 0.1	0.5 ± 0.0	0.5 ± 0.1	1.0 ± 0.1	1.5 ± 0.2	—
P05090	Apolipoprotein D^#^	0.3 ± 0.0	8.7 ± 0.4	11.2 ± 0.6	1.8 ± 0.3	9.0 ± 2.3	3.4 ± 0.5	5.7 ± 0.4
P02649	Apolipoprotein E^#^	0.5 ± 0.0	2.2 ± 0.5	0.4 ± 0.2	3.2 ± 1.5	5.6 ± 2.1	11.0 ± 3.0	7.6 ± 1.3
P10909	Clusterin^#^	0.5 ± 0.0	9.4 ± 1.0	6.9 ± 1.6	2.4 ± 0.6	8.6 ± 1.7	12.2 ± 2.9	22.8 ± 11.2
	***Immunoglobulins (% of total protein)***	**23.1** ± **1.4**	**6.7** ± **0.3**	**8.1** ± **0.8**	**4.5** ± **1.3**	**2.9** ± **0.6**	**4.2** ± **0.4**	**0.0** ± **0.0**
P01876	Ig α-1 chain C region	1.9 ± 0.1	*	*	0.2 ± 0.1	0.5 ± 0.1	—	—
P01857	Ig γ-1 chain C region	8.5 ± 0.2	2.5 ± 0.1	3.1 ± 0.3	1.4 ± 0.5	1.4 ± 0.2	4.2 ± 0.4	*
P01859	Ig γ-2 chain C region	3.9 ± 0.1	1.7 ± 0.2	2.0 ± 0.3	1.1 ± 0.3	1.0 ± 0.3		*
P01860	Ig γ-3 chain C region	7.8 ± 0.3	2.5 ± 0.1	3.0 ± 0.2	1.4 ± 0.5	*	*	—
P01834	Ig κ chain C region	1.0 ± 0.7	*	*	0.5 ± 0.0	*	*	*
	***Blood coagulation (% of total protein)***	**0.7** ± **0.0**	**4.1** ± **0.5**	**3.8** ± **0.7**	**1.8** ± **0.3**	**2.2** ± **0.4**	**0.0** ± **0.0**	**0.0** ± **0.0**
P01009	α_1_-antitrypsin	0.7 ± 0.0	0.5 ± 0.2	*	1.0 ± 0.2	*	*	*
P02671	Fibrinogen α chain	*	0.4 ± 0.0	0.4 ± 0.0	0.1 ± 0.0	*	*	—
P02751	Fibronectin^#^	*	0.9 ± 0.0	1.2 ± 0.3	0.2 ± 0.1	0.7 ± 0.1	*	—
P23142	Fibulin-1	*	1.4 ± 0.1	1.3 ± 0.1	0.3 ± 0.0	1.2 ± 0.2	—	—
P04196	Histidine-rich glycoprotein	*	0.4 ± 0.1	0.6 ± 0.2	0.1 ± 0.0	*	—	—
P00734	Prothrombin	*	0.4 ± 0.1	0.3 ± 0.0	0.2 ± 0.0	0.4 ± 0.2	*	—
	***Complement system (% of total protein)***	**0.1** ± **0.0**	**1.6** ± **0.3**	**0.9**	**0.1**	**0.9** ± **0.2**	**2.2** ± **0.6**	**0.0** ± **0.0**
P09871	Complement C1s subcomponent	*	0.2 ± 0.0	*	0.2 ± 0.0	0.7 ± 0.2	—	—
P01024	Complement C3	0.1 ± 0.0	0.3 ± 0.1	0.3 ± 0.1	0.8 ± 0.2	*	—	—
P0C0L4	Complement C4-A	*	0.3 ± 0.1	*	0.3 ± 0.0	0.6 ± 0.2	*	—
P08603	Complement factor H	0.0 ± 0.0	0.8 ± 0.1	0.9 ± 0.1	0.1 ± 0.0	*	—	—
	***CSF specific proteins (% of total protein)***	**4.7** ± **0.3**	**8.9** ± **0.8**	**12.3** ± **2.1**	**2.0** ± **0.3**	**3.9** ± **0.2**	**7.1** ± **1.4**	**0.0** ± **0.0**
P51693	Amyloid-like protein 1	*	2.8 ± 0.2	3.4 ± 0.7	0.6 ± 0.0	2.0 ± 0.1	*	—
O94985	Calsyntenin-1^#^	*	1.2 ± ± 0.0	1.8 ± 0.8	0.4 ± 0.2	1.2 ± 0.1	3.1 ± 0.4	—
P01034	Cystatin-C	4.6 ± 0.3	3.9 ± 0.4	6.0 ± 0.6	0.9 ± 0.0	*	*	*
Q9UHG2	ProSAAS	0.1 ± 0.0	1.0 ± 0.1	1.1 ± 0.1	0.2 ± 0.0	0.7 ± 0.0	4.0 ± 0.9	—
	***Other proteins (% of total protein)***	**24.5** ± **1.7**	**46.1** ± **2.3**	**54.1** ± **14.4**	**75.6** ± **2.8**	**54.8** ± **7.5**	**75.1** ± **13.1**	**68.0** ± **14.6**
P02765	α_2_-HS-glycoprotein	0.2 ± 0.0	1.1 ± 0.0	1.9 ± 0.6	0.7 ± 0.2	3.6 ± 0.7	*	—
Q9UBP4	Dickkopf-related protein 3	0.3 ± 0.0	7.2 ± 0.3	5.5 ± 2.2	1.5 ± 0.4	3.9 ± 1.5	10.9 ± 1.0	—
Q12805	EGF-containing fibulin-like extracellular matrix protein 1	*	0.6 ± 0.2	1.0 ± 0.3	0.2 ± 0.1	0.7 ± 0.1	*	—
P06396	Gelsolin^#^	0.4 ± 0.0	2.9 ± 0.1	3.5 ± 0.6	0.8 ± 0.1	1.9 ± 0.2	5.0 ± 0.9	—
P20774	Mimecan	*	0.5 ± 0.0	0.6 ± 0.2	0.2 ± 0.1	0.4 ± 0.1	2.1 ± 0.9	*
P02768	Serum albumin^#^	23.6 ± 1.7	27.8 ± 1.0	33.8 ± 8.8	69.7 ± 1.6	38.8 ± 3.5	49.3 ± 9.1	63.6 ± 14.3
Q14515	SPARC-like protein 1	*	1.2 ± 0.1	1.4 ± 0.0	0.4 ± 0.1	0.8 ± 0.4	*	—
P02766	Transthyretin^#^	*	3.0 ± 0.5	4.0 ± 1.3	1.1 ± 0.2	2.4 ± 0.2	4.3 ± 1.1	4.4 ± 0.3
P02774	Vitamin D-binding protein	0.0 ± 0.0	0.7 ± 0.0	1.1 ± 0.0	0.8 ± 0.1	1.4 ± 0.8	—	—
P04004	Vitronectin	*	1.1 ± 0.0	1.3 ± 0.3	0.2 ± 0.1	0.8 ± 0.1	3.6 ± 0.2	—

Proteins constituting > 0.5% of the total composition, for at least one fibril system are displayed. Respective fibril peptides have been removed from the quantification. The data represents nine technical replicas with standard deviations.

*Identified, but not quantified. —Not identified in any of the samples. ^#^Significant band in gel analysis.

The composition of the amyloid interactomes was highly influenced by the body fluid type even though it clearly differed from the composition of the fluid type itself. One such example was serum albumin that constitutes the major component of both plasma and CSF (53.1% and 23.6% respectively). In our case, serum albumin showed the biggest difference in contributions to individual fibril interactomes and the biggest difference between fibril interactomes in the two fluids. In plasma, the level of serum albumin in the fibril interactomes was significantly lower than the original relative concentration as it ranged from 2.5% for Ab1 to 19.9% for glucagon. In contrast, the contribution was increased in the fibril interactomes to 27.8–69.7% in CSF compared to the original concentration.

Vitronectin is another protein that exposed significant differences between the two body fluids. In plasma, the relative concentration is 0.2% but the fibril-associated relative percentage is increased to 3.7–17.1% with glucagon and the Aβ fibrils representing the lowest and highest association, respectively. In CSF, the relative concentration is below the detection limit and the contribution to the amyloid interactome is in the range of 0.2–3.6% with αSN fibrils demonstrating the highest relative amount of bound vitronectin.

Immunoglobulins contributed little to the fibril interactomes in both fluids (4.5–5.5% in plasma and 2.9–8.1% in CSF), despite the high relative concentration of these proteins in both fluids (22.9% in plasma and 23.1% in CSF). Apolipoproteins, however, has significantly increased contributions to the fibril interactomes compared to the original fluid concentration. In plasma, the relative concentration was 2.7%, but the contribution ranged from 22.5 to 45.1% in the fibril interactomes. In general, ApoA-I showed the highest contribution (8.8–14.9%), while ApoC-III showed the highest specificity directed towards the αSN fibrils (11.7%). In CSF, the relative concentration of apolipoproteins was 1.7% and the contribution to the fibril interactomes ranged from 11.2% to 30.8%. Compared to the interactome in plasma, ApoA-I contributed less, while ApoD and clusterin contributed significantly more to the fibril interactomes.

The proteins involved in blood coagulation and in the complement system are low-abundant in CSF compared to plasma. This was mirrored by the relative contributions to the fibril interactomes in the two body fluids. In plasma, proteins involved in blood coagulation contributed with 30.1 to 41.1% of the interactomes compared to 0.0 to 4.1% in CSF. For both fluids, the proteins were more abundant in the interactomes compared to the original fluids. Similarly, the proteins involved in the complement system were more concentrated in the interactomes than in the original fluids. In plasma, the contribution ranged from 6.6 to 14.4% while in CSF it ranged from 0.0 to 2.2%.

For the non-conventional fibril-like FAS4 AD aggregates, we observed less protein binding and a different composition of the bound proteins when compared to the fibril systems. In plasma, the relative contribution of aggregate-associated proteins involved in blood coagulation (6.6%) and the complement system (2.7%) was much lower than for the fibril systems, while the Immunoglobulins showed an increased relative contribution (23.1% for FAS4 AD versus 4.5–5.5% for the other fibril systems). In absolute terms, the amount of bound Immunoglobulins was similar for the FAS4 AD aggregates and the fibril systems, but all other fibril-associated proteins were drastically reduced. In CSF, only five proteins were quantified as interaction partners with FAS4 AD of which serum albumin (63.6%) and clusterin (22.8%) were the main contributors.

For a quantitative comparison of the protein amount bound to the fibril surface (protein:fibril ratio) we plotted the absolute ion intensity for the different protein groups (apolipoproteins, blood coagulation, immunoglobulins, complement system, CSF-specific, and Other proteins) as a function of the fibril system (Figure S11, S12). Intensity differences directly reflect changes in the protein:fibril ratio because the fibril amount was kept constant. For most protein groups, we observed higher ion intensities for Ab1, Ab2, glucagon, C-36, and αSN compared to the FAS4 AD and empty controls. Additionally, we observed slightly elevated intensities from the glucagon and C-36 fibrils compared to the other fibril systems. The total ion intensity decreased for all protein groups in CSF compared to plasma. This protein association decrease likely reflects the lower protein:fibril incubation ratio (50:1 in plasma versus 4:1 in CSF). However, the associated protein decrease was particularly evident for the αSN fibril system, which suggests system-specific differences as well. The observations are all consistent with the presented SDS-PAGE analyses.

## Discussion

Small amyloid-binding molecules are thought to bind to amyloid fibril surface grooves originating from the repetitive β-sheet arrangement and the specific side chain arrangement^45,48^. They have attracted significant attention because of their diagnostic and therapeutic potential but can also be employed as low-resolution probes for structural differences between amyloid species. We used ThT, FSB, ANS, and Orange-G to map the interaction potential of our different fibril systems.

Cross-strand ladders on the fibril surface caused by the repetitive side-chain arrangement, are believed to be the interaction site for ThT^40^. The fluorescence arises from a binding-induced limitation in ThT’s internal rotation^49^, and by comparing the florescence intensities we confirmed different surface properties of our fibril systems. ThT fluorescence intensity displayed up to a 10-fold difference between fibril types (C-36 fibrils versus glucagon fibrils), which was not correlated to the number of ThT binding sites at the surface (Fig. 2). The absolute binding of ThT, FSB, and Orange-G served to further illustrate the differential recognition of tracers to amyloid surfaces. The highly similar profile for tracer binding to the two Aβ polymorphs suggests that primary sequence is a major determinant for recognition rather than the fibril morphology. Considering the several published structures of two- and three-fold symmetric Aβ polymorphs^20,50^, we cannot exclude minor additional effects of the exact β-sheet organization on ligand recognition.

We had clear indications by fibril morphology and molecular tracer binding that fibrillar surfaces harbour structural differences. Such differences were expected to influence protein binding in plasma and CSF. However, both gel analyses and quantitative mass spectrometry gave very similar results regarding associated protein amount and composition for all fibril systems (Fig. 3). The gel band identification and the quantitative MS/MS analysis revealed many amyloid interaction partners, most of which have previously been described in the literature^25,51–64^. These included clusterin, ApoE, fibrinogen, and Dickkopf-related protein 3. The identification of previously reported amyloid interaction partners validated our model system and comparative approach. The shared amyloid interactome across diverse fibrillar structures establishes the existence of a generalized response to the amyloid surface in body fluids that has not previously been demonstrated. Our comprehensive list of amyloid-associated proteins in CSF and plasma enables future studies of the role of this interactome in amyloid deposition and toxicity. By comparing the amyloid interactome with the protein composition in the original body fluids, we conclude that the amyloid interactome did not arise from a general aggregation of soluble proteins. Furthermore, the lack of co-aggregated proteins with the TCA-induced aggregates of chymotrypsinogen revealed that the interactome is specific for the amyloid fold. The aggregates of FAS4 AD appeared to have similar secondary structures as the fibril systems when studied by FTIR but did otherwise not resemble fibril structures. As a result, the FAS4 AD aggregates gave rise to some co-aggregation of other proteins, but the composition was significantly different when compared to the fibrillar samples. Combined, our results point towards the existence of a general amyloid interactome which recognise fibrils with the right amyloid secondary and tertiary structure.

We found a clear enrichment of other amyloidogenic proteins in the amyloid interactome, including ApoA-I^51,65^ ApoA-II^51^, ApoA-IV^27^, Ig light- and heavy-chains^27^, transthyretin^29^, and Cystatin C^66^. Their specificity to elongated fibrils may be explained by the fact that exposure to a fibril surface can push aggregation-prone proteins to accumulate, potentially by cross-seeding^67,68^.

Amyloid proteins accumulate in various tissue compartments and are exposed to either plasma-borne or CSF proteins, depending on their primary area of manifestation^4^. Both relevant biological fluids share several proteins but differ substantially in their composition. We identified plasma and CSF-specific protein enrichment profiles at the fibril surface which had several overlapping protein identities, however, large variations existed in the relative interactome contribution. Such examples included ApoA-I, ApoD, and vitronectin. Importantly, the variations between the amyloid interactome in the two body fluids were significantly bigger than any observed variation between two fibril systems in the same medium. The origin of these variations should be found in the overall protein composition and concentration that affect the binding equilibrium in each biological fluid. In CSF, proteins involved in blood coagulation and the complement system are low-abundant and consequently these proteins were mostly absent in the CSF amyloid interactome. In contrast, the relative contribution from serum albumin was increased significantly in CSF compared to the plasma amyloid interactome as were the contributions from CSF-specific proteins amyloid-like protein 1, calsyntenin-1, Cystatin-C, and ProSAAS.

Among all our observations, the poor level of protein interaction with αSN fibrils in CSF is striking. The fibril-specific differences that render the αSN fibril surface less susceptible to co-aggregation in CSF could be linked to its elevated ANS fluorescence level compared to the other fibril types although more research is needed to understand this relationship further.

A few proteins showed selectivity towards certain fibril systems when combining observations in the gel analysis (Fig. 3) and the relative compositions (Tables 1 and 2). In plasma, ApoA-II showed elevated binding to glucagon fibrils, ApoC-III to C-36 and αSN fibrils, while vitronectin bound predominantly to the Aβ and αSN fibrils. Additionally, we observed a general preference for serum albumin towards glucagon fibrils. Although the biological consequences of these amyloid-specific systems remain unknown, it is relevant to note the high abundance of fibril-associated chaperones (ApoE^69^ and clusterin (ApoJ^70^)) found for many of our fibril systems. Interestingly, the high abundance of these chaperones in amyloid deposits has been suggested to attenuate *in vivo* fibril formation^71–73^, which is supported by clusterin’s *in vitro* inhibitory effect on the primary and secondary nucleation of Aβ fibrillation^74^. A recent study of the three chaperones DNAJB6, Ssa1, and proSP-C Brichos confirms the role of chaperones in modulation of fibrillation by targeting primary, secondary nucleation, and fibril elongation^75^. Our results suggest an intrinsic affinity for chaperones to the amyloid fold where they may act as protectants to prevent uncontrolled aggregation. Such a chaperone activity in complex media has been observed for Aβ42 fibrillation in CSF^76^ and could be a general feature of the body’s response to an amyloid load.

The generalized phenomenon of broad protein association to the repetitive amyloid surface is paralleled by protein coronas associated with nanoparticles of underlying repetitive molecular arrangements. In fact, most of the highly abundant proteins in the NP coronas were also present in the amyloid interactome albeit only constituting a minor fraction. ApoB-100 is one such example; it is the major component in the NP coronas^77^ but only contributes 0.4% to the amyloid interactome in plasma.

Changing the NP size or the surface charge clearly affects the protein composition of the coronas^78^ but variations to the same degree were not observed for the fibril interactomes despite differences in fibril width, peptide length, and overall charge of the peptides (Aβ -3, Glucagon, 0, C-36 + 3, αSN -9, FAS4 AD −2). Although we do suspect that the minor fibril-specific differences are correlated to the surface properties, the generalized picture that emerges is that of a dominant proteins-association property based on the architecture of the amyloid fold.

In conclusion, the introduction of elongated fibrils into plasma or CSF triggers a specific response by the co-aggregation of soluble proteins. The composition of co-aggregated proteins was only slightly influenced by the fibril structure and as a result, we established a general amyloid interactome for the two body fluids. Based on the differences between the amyloid interactome in plasma and CSF, we conclude that the location of amyloid deposits appears to have a greater influence on the composition of the fibril-associated proteins than does the exact nature or structure of the amyloidogenic fibrillar peptides and aggregates.

## Materials and Methods

### Materials

Outdated human plasma was obtained from Aarhus University Hospital, Skejby, Denmark. Cerebrospinal fluid (CSF), also obtained from Aarhus University Hospital, was extracted from three healthy donors and pooled to minimize biological variation. The solution was clear with no visible indication of blood contamination. All work was carried out in accordance with the Declaration of Helsinki (59th amendment). Ethics approval was granted by the Research Ethics Committee of the Central Denmark Region (De Videnskabsetiske Komitéer for Region Midtjylland). All participants provided written informed consent.

(E,E)-1-fluoro-2,5-bis-(3-hydroxycarbonyl-4-hydroxy)styrylbenzene (FSB) was synthesized^79^ and provided by prof. Troels Skrydstrup at iNANO and Department of Chemistry, Aarhus University. Thioflavin T (ThT) and orange-G were purchased from Sigma Aldrich. Thioflavin T was purified by several rounds of recrystallization in Milli-Q water (Millipore Corporation), while Orange-G was used without further purification.

Buffer compositions were as follows; phosphate buffer (10 mM sodium phosphate, pH 7.4), PBS (10 mM sodium phosphate, 137 mM NaCl, pH 7.4) and Glycine buffer (50 mM Glycine, pH 2.5).

### Fibril preparation

Synthetic amyloid β peptide 1–40 was purchased from Caslo Aps (Lyngby, Denmark). Dry Aβ peptide powder was dissolved in DMSO to a concentration of 15 mg/mL, diluted five times in phosphate buffer and incubated at room temperature at quiescent conditions for two days. A seeding protocol was adopted with inspiration from Petkova *et al*.^37^. In short, a solution of parent fibrils was sonicated vigorously and diluted into phosphate buffer before addition of monomeric Aβ peptide from a 15 mg/mL peptide stock in DMSO to 0.5 mg/mL (125 μM) Aβ concentration (5–10% seeds wt%). After 24 h, 10% of the volume was sonicated and mixed with the remaining sample. After additional four days, the fibrils were collected by centrifugation (17,000 g, 30 min), washed once and re-suspended in the same volume of phosphate buffer. Homogeneous fibrils were obtained after five rounds of seeding, and these fibrils were used as seeds for the Ab1 fibrils included in this study.

A second protocol was adopted to prepare structurally different Aβ fibrils. A 15 mg/mL peptide stock in DMSO was diluted 30 times into phosphate buffer and left at room temperature with gentle agitation for three days. Four generations of seeding resulted in a homogenous stock of fibrils that were used as seeds for the Ab2 fibrils included in this study.

Expression and purification of the FAS4 A546D mutant protein was performed as previously described^80^. The protein was concentrated to 0.12 mg/mL in PBS buffer and fibrillated at 37 °C and 900 rpm shaking for 7 days. The resulting fibrils were collected by centrifugation (17,000 g, 30 min), washed once and re-suspended in the same volume of phosphate buffer.

The α-synuclein protein (αSN) was expressed and purified using literature methods^81,82^. Fibrillation was performed according to established methods^83^. Dry αSN protein was dissolved in PBS buffer to 2 mg/mL (138 μM) concentration and fibrillated at 900 rpm shaking and 37 °C for 3 days. The resulting fibrils were collected by centrifugation (17,000 g, 30 min), washed once and re-suspended in the same volume of PBS buffer.

Pharmaceutical grade glucagon (>98.9% purity) was kindly provided by Novo Nordisk A/S (Bagsværd, Denmark). Dry glucagon peptide was dissolved in Glycine buffer to 0.5 mg/mL and left for fibrillation with 500 rpm shaking at 37 °C for two days according to established methods^16^. The resulting fibrils were collected by centrifugation (17,000 g, 30 min), washed once and re-suspended in the same volume of phosphate buffer.

The 36 residue C-terminal of α_1_-antitrypsin (C-36) was expressed and purified as previously described^84^. A solution of C-36 peptide in miliQ water (235 μM) was diluted into PBS buffer yielding a final concentration of 0.2 mg/mL (50 μM). The sample was left for fibrillation at 300 rpm shaking and 37 °C for three days. The resulting fibrils were collected by centrifugation (17,000 g, 30 min), washed once and re-suspended in the same volume of phosphate buffer.

In all cases, the appearance of the fibrils was checked by TEM and fibrils were stored at either 4 °C with 0.02% sodium aside in the buffer or flash frozen in liquid nitrogen and stored at −20 °C.

### Trichloroacetic acid (TCA) precipitation of chymotrypsinogen

Ice cold TCA was added to dissolved chymotrypsinogen (Amersham) to a final concentration of 20% (v/v) and incubated on ice for one hour. The entire protein content was precipitated, and the sample was centrifugated for 30 min. at 17,000 g. The pellet washed once in 96% ethanol before it was lyophilized and resuspended in PBS.

### Transmission Electron Microscopy (TEM) analysis

Small aliquots of the fibril samples were washed once in miliQ water and then deposited on a glow-discharged carbon-coated grid. The sample was briefly washed in H_2_O, blotted dry, and stained with 1% uranyl acetate in water for one minute. TEM images were collected using a Tecnai G2 spirit electron microscope, operated at 90 KeV with a LaB6 filament.

The width of the individual fibrils was measured using the ImageJ software^85^ and counted in bins of 0.5 nm. Average fibril width and distribution was extracted by modelling the count data to a normal distribution function.

### Fourier-transform infrared spectroscopy

FTIR analysis was conducted on a Tensor 27 (Bruker) FTIR spectrophotometer equipped with a DTGS Mid-infrared detector and a Golden Gate single reflection diamond attenuated total reflectance cell (Specac). Approximately 4 μg of aggregated protein was placed and dried on the ATR crystal using dry nitrogen. Spectra were recorded from 4000–1000 cm^−1^ using a resolution of 2 cm^−1^ and 64 accumulations. Data was corrected for background and atmospheric interference and normalized to the peak intensity.

### Fibril quantification

The protein amount for each fibril stock was evaluated by Tricine gels and Coomassie blue staining. In addition, the fibrils stock concentrations were evaluated by reverse-phase chromatography (RP300, Brownlee, 220 × 4.6 mm) of Guanidinium chloride-solubilized samples using the UV/VIS absorbance at 205 nm and calculated specific extinction coefficients at 205 nm for each protein^86^.

### Quantification of molecular tracer stocks by NMR

Dry powder of ThT, Orange-G, and FSB was dissolved in d_6_-DMSO and transferred to NMR tubes together with either 6 or 12 mM ethanol as internal standard. Liquid-state ^1^H-NMR spectra were acquired on a Bruker 400 MHz NMR spectrometer (Bruker Rheinstetten, Germany) with 16 scans and 3 sec relaxation delay. The concentration was determined based on peak integrals and the stocks were diluted to 10 mM in DMSO.

### Determination of the binding capacity

Fibril material (10 μg) was mixed with ThT, FSB, or orange-G (2 nmol) in 100 μL phosphate buffer (0.2% DMSO) and incubated for one hour. Control samples (without fibril material) were prepared similarly. Fibrils and fibril-bound ligand were pelleted by centrifugation (17,000 g, 30 min) and the supernatant (90 μL) was transferred to a 96 well half-area plate (Corning 3881). The ligand absorbance (ThT 412 nm, FSB 350 nm, orange-G 480 nm) was measured using a FLUOstar Omega plate reader (BMG Labtech) blanked against buffer and baseline corrected to the absorbance at 700 nm.

### Fibril titration with ThT

Fibril material (7.5 μg) was set up in 100 μL phosphate buffer in a 96 well half-area NBS plate (NUNC/Terma). Stepwise ThT titration was conducted from 0.01 to 11 μM with 100 sec mixing (300 rpm) before each measurement using a FLUOstar Omega plate reader (excitation at 450 nm, emission at 485 nm). The relation between ThT concentration and fluorescence intensity was fitted to a single site binding model, I = (I_max_ * C)/(K_d_ + C), for glucagon, αSN, and C-36 fibrils, whereas the signal from Ab1 and Ab2 fibrils was fitted to a two-step binding model, I = (I_max1_ * C)/(K_d1_ + C) + (I_max2_ * C)/(K_d2_ + C).

### Fibril titration with ANS

Fibril material (2.5 μg) was set up in 50 μL phosphate buffer in a 96 well half-area NBS plate (NUNC/Terma). Stepwise ANS titration was conducted from 20 to 167 μM with 100 sec mixing (300 rpm) before each measurement using a FLUOstar Omega plate reader (excitation at 450 nm, emission at 485 nm).

### Fibril incubation with plasma and CSF

Plasma and CSF solutions were centrifuged (40,000 g, 20 min) to remove any aggregates. The protein concentration was determined using 280 nm absorbance assuming 1 Abs = 1 mg/mL. Samples with fibril material (20 μg), plasma (500 μg) or CSF (40 μg) proteins were prepared in 200 μL PBS buffer with 5 mM EDTA. Control samples (without fibril material) were prepared similarly. All samples were incubated at room temperature for one hour under gentle agitation. Fibrils and fibril-bound proteins were pelleted by centrifugation at 17,000 g for 30 minutes. The supernatant (175 μL) was removed and the aggregate was re-suspended in 175 μL PBS and pelleted again before further preparation for gel analysis and spin-filter assisted digest for quantitative MS/MS.

### Gel analysis of the amyloid interactome

The fibril-protein pellets were washed once in 1 M NaCl and twice in PBS. SDS sample buffer containing 50 mM DTT was added and all samples were boiled for 5 minutes before loaded onto a SDS 10–15% acrylamide gel^87^. Triplicate samples were analysed on three different gels. Relevant gel bands were in-gel digested with trypsin^88^, and the resulting peptides were micro-purified using Emporer C18 column material packed in P10 pipette tips (VWR).

### Sample preparation for quantitative MS/MS

The fibril-protein pellets were transferred to spin-filters (0.22 μm), washed twice in PBS (100 μL) and once in 1 M NaCl (100 μL). The fibrils and fibril-bound proteins were dissolved by one-hour incubation in 100 μL 6 M guanidinium chloride followed by elution from the spin-filter. The eluted proteins were loaded onto a 3 kDa MWCO filter unit for filter-assisted unfolding, reduction, alkylation, and trypsin digestion^89^. In brief, samples were washed twice in 8 M urea, once in 8 M urea with 50 mM DTT, once in 8 M urea with 50 mM iodoaceteamide, and twice in 8 M Urea. The filter units were dried by centrifugation (14,000 g in 30 minutes) after every addition. Trypsin digestion was carried out with 0.25 μg sequencing grade trypsin from Sigma Aldrich in 50 mM ammonium bicarbonate overnight at 37 °C. The tryptic peptides were collected by centrifugation, acidified with TFA (0.1%) and micro-purified using POROS R2 column material (Applied Biosystems) packed in P200 pipette tips (Sarstedt).

### In-solution digest of CSF and plasma for quantitative MS/MS

One µL CSF and plasma were lyophilized and proteins were solubilized in 8 M urea with 100 mM ammonium bicarbonate. The sample were reduced and alkylated with 10 mM DTT and subsequently 30 mM iodoacetamide (IAA). To neutralized excess IAA, DTT was added to a final concentration of 35 mM. The samples were digested with trypsin (1:50 w/w) for 16 hrs at 37 C. Reaction was stopped by acidifying the sample with formic acid. Peptides were micropurified prior to MS analysis.

### LC-MS/MS analysis

LC-MS/MS analyses were performed on an EASY-nLC II system (Thermo Fisher Scientific) connected to a TripleTOF 5600+ mass spectrometer (Sciex) equipped with a NanoSpray III source (Sciex) and operated under Analyst TF 1.6.0 control.

The micro-purified tryptic peptides were dissolved in 0.1% formic acid, injected and trapped on an in-house packed trap column (2 cm × 100 μm I.D) with RP ReproSil-Pur C18-AQ 3 μm resin (Dr. Maisch GmbH). Peptides were eluted from the trap column and separated on a 15 cm analytical column (75 μm i.d.) packed in-house in a pulled emitter with RP ReproSil-Pur C18-AQ 3 μm resin (Dr. Maisch GmbH). Elution from the analytical column was performed with a linear gradient from 5% to 35% phase B (90% acetonitrile with 0.1% formic acid) over 20 min for protein identification and 50 min for quantitative analysis, respectively.

### Protein identification from gel bands

The collected MS files were converted to Mascot generic format (MGF) using AB SCIEX MS Data converter beta 1.1 (AB SCIEX) and the “Protein MGF” parameters. Generated peak-lists were searched in the Swiss-Prot (v. 2015_08) Homo sapiens database using Mascot 2.5.0 (Matrix science, London, UK). Propionamide modifications of cysteines were fixed, methionine oxidation was tolerated, and one trypsin miss-cleavage was allowed for the protein identification.

The instrument setting was set to ESI-QUAD-TOF and the search was performed with a 10 ppm and 0.2 Da mass tolerance of the precursor and product ions respectively. All searches were adjusted to a 1% FDR at the peptide level. The data were imported and processed using MS Data Miner v. 1.3.0^90^.

General laboratory contaminants were excluded before the highest scoring proteins were assigned to the gel-bands. In cases with more than one high-scoring protein, assignment of two proteins to a gel-band was allowed when the second protein had a score and emPAI value above 50% of the highest scoring protein.

### Quantitative MS/MS

All samples were analysed three times, yielding nine MS/MS experiments for each fibril for both plasma and CSF. For the area-based extracted ion chromatogram (XIC) quantification, the acquisition method was set up as an information-dependent acquisition (IDA) experiment collecting up to 25 MS/MS spectra in each 1.6 sec cycle using an exclusion window of 6 sec.

The area-based XIC quantification was done in Mascot Distiller 2.5.1.0 (Matrix Science) using the following inclusion criteria for the default average [MD] quantification protocol: A 1% FDR threshold, three or more unique peptides observed per protein, a matched rho of 0.8, an XIC threshold of 0.3, and an isolated precursor threshold of 0.7 Da.

### Quantitative MS/MS data analysis

XIC quantification was based on the average ion intensity of the three most abundant peptides for each protein. Only proteins quantified in at least four out of nine MS/MS experiments per fibril were included in the quantification. The total ion scores were visualized after grouping the proteins according to function (Figure S11).

For each MS/MS experiment, the ion intensities of the individual proteins were normalized to the total ion intensity of that MS/MS experiment to achieve a relative protein composition. The contribution of each protein was averaged over all nine MS/MS experiments (Tables 1 and 2).

## Supplementary information

Supp_information_SREP-18-03543

## Data Availability

The data sets and analyses generated in the current study are available from the corresponding author upon request.
