# Supplementary material for: Conservation of the Amyloid Interactome Across Diverse Fibrillar Structures
Source: Sci Rep. 2019 Mar 7;9:3863. doi: 10.1038/s41598-019-40483-z (PMC6405930; doi:10.1038/s41598-019-40483-z)
Supplement: Supplementary file 1 — Supp_information_SREP-18-03543 [file 41598_2019_40483_MOESM1_ESM.docx]

**Conservation of the Amyloid Interactome Across Diverse Fibrillar Structures.**

Dennis Wilkens Juhl^1,2,3^, Michael Wulff Risør^1,2^, Carsten Scavenius^1,2^, Casper Rasmussen^2^, Daniel Otzen^1,2^, Niels Chr. Nielsen^1,3^, and Jan J. Enghild^1,2,*^.

^1^Interdisciplinary Nanoscience Center (iNANO), Aarhus University, DK-8000, Aarhus, Denmark.

^2^Department of Molecular Biology and Genetics, Aarhus University, Aarhus, Denmark.

^3^Department of Chemistry, Aarhus University, Aarhus, Denmark.

* jje@mbg.au.dk


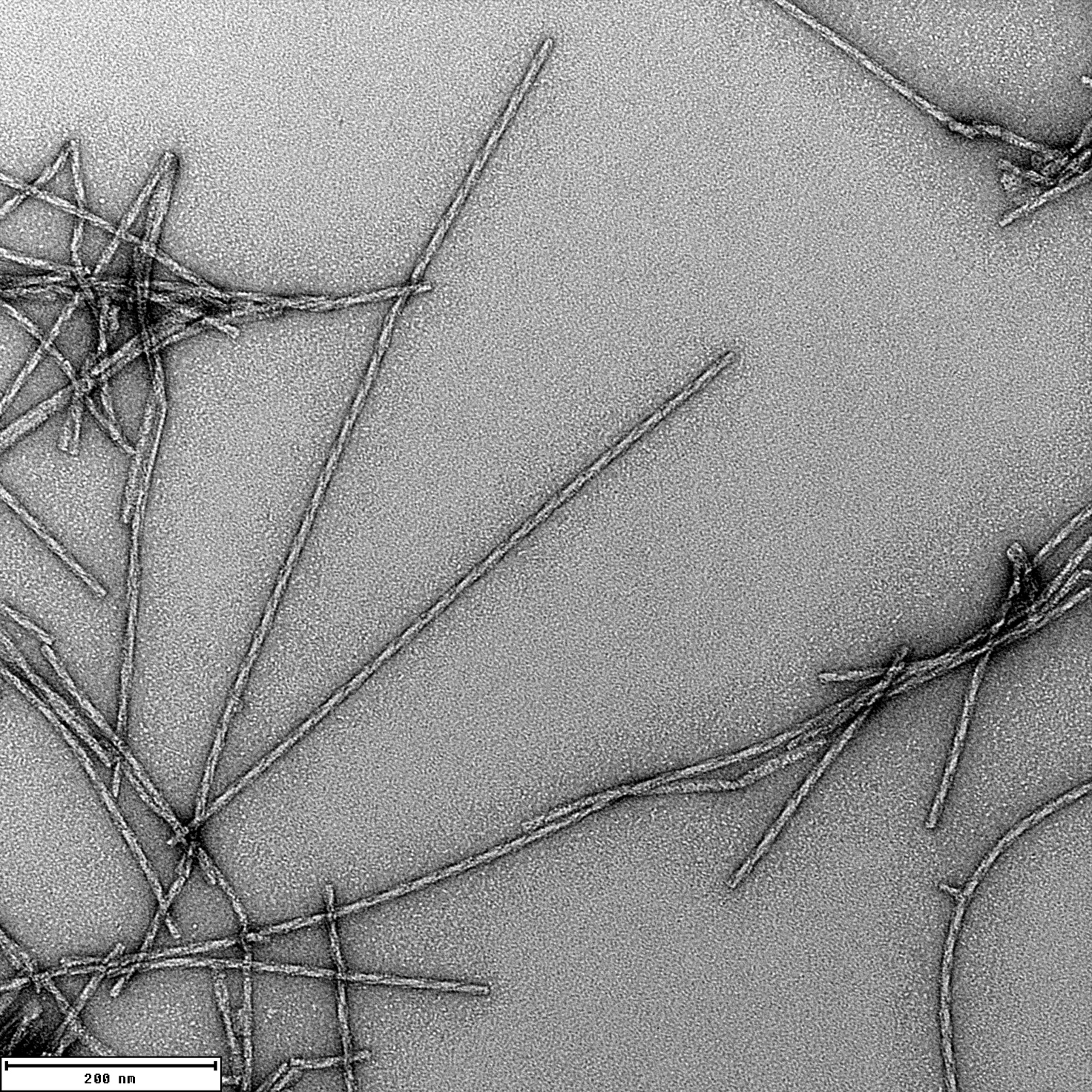


**Figure S1**. Full-scale TEM image of the Ab1 fibrils illustrated in figure 1.


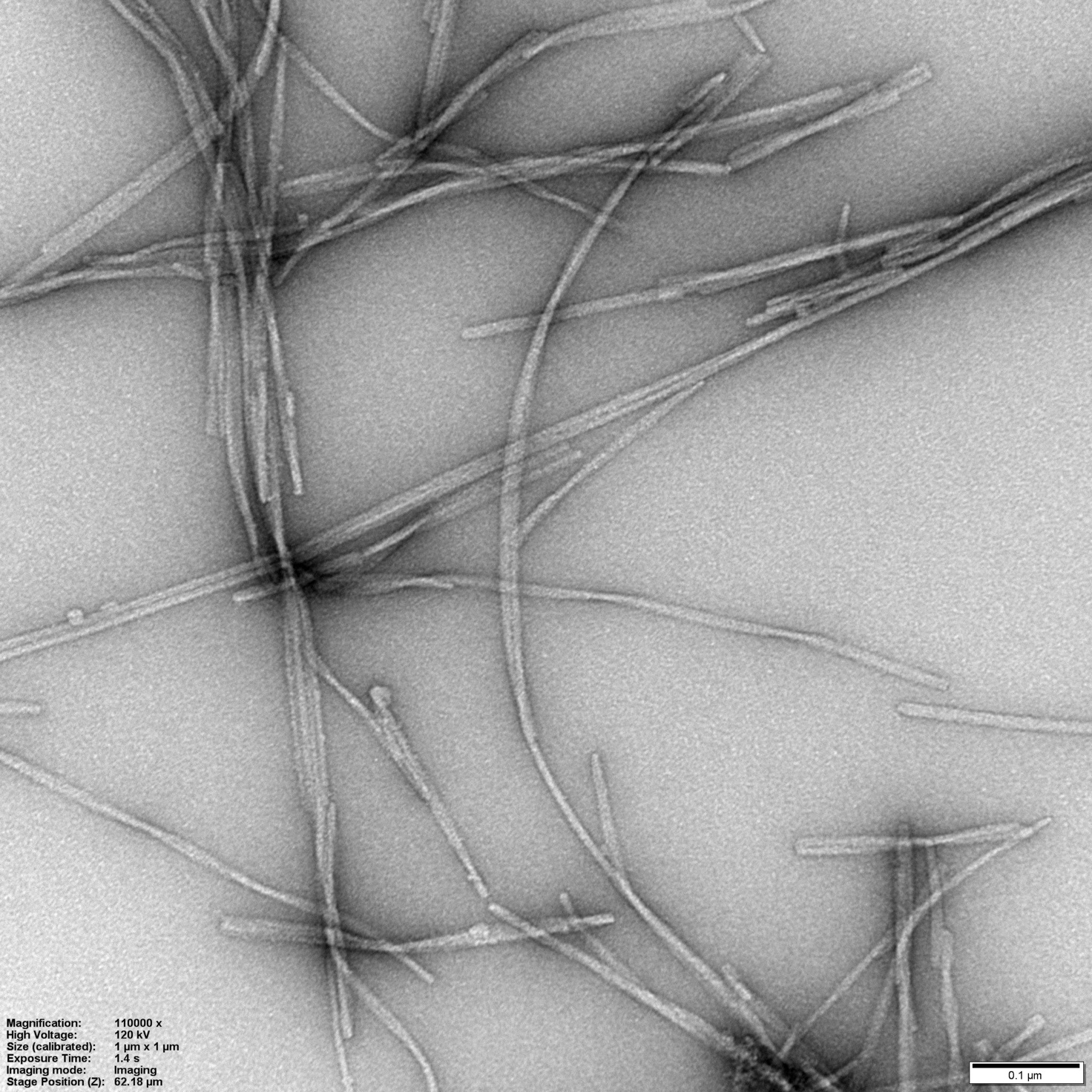


**Figure S2**. Full-scale TEM image of the Ab2 fibrils illustrated in figure 1.


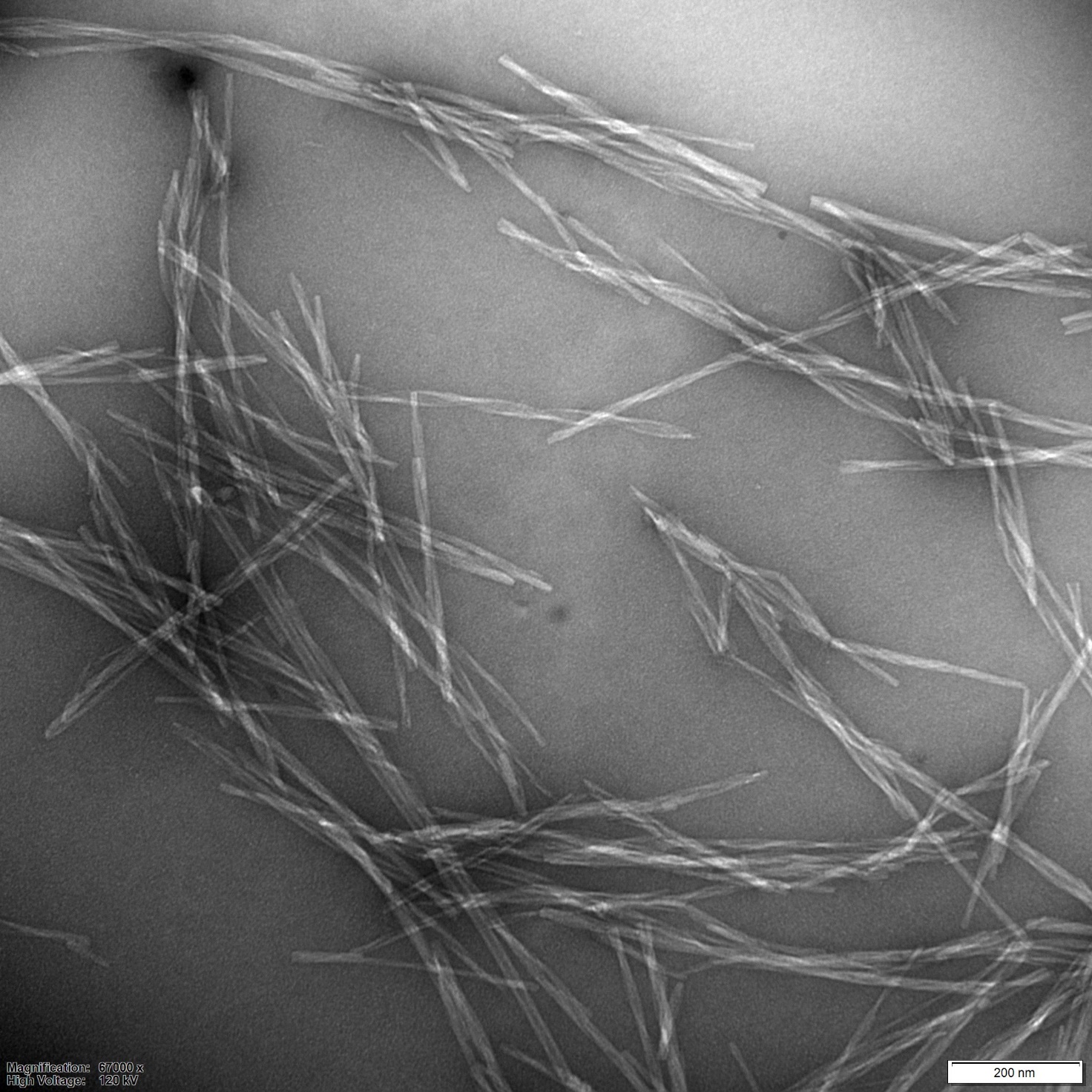


**Figure S3**. Full-scale TEM image of the Glucagon fibrils illustrated in figure 1.


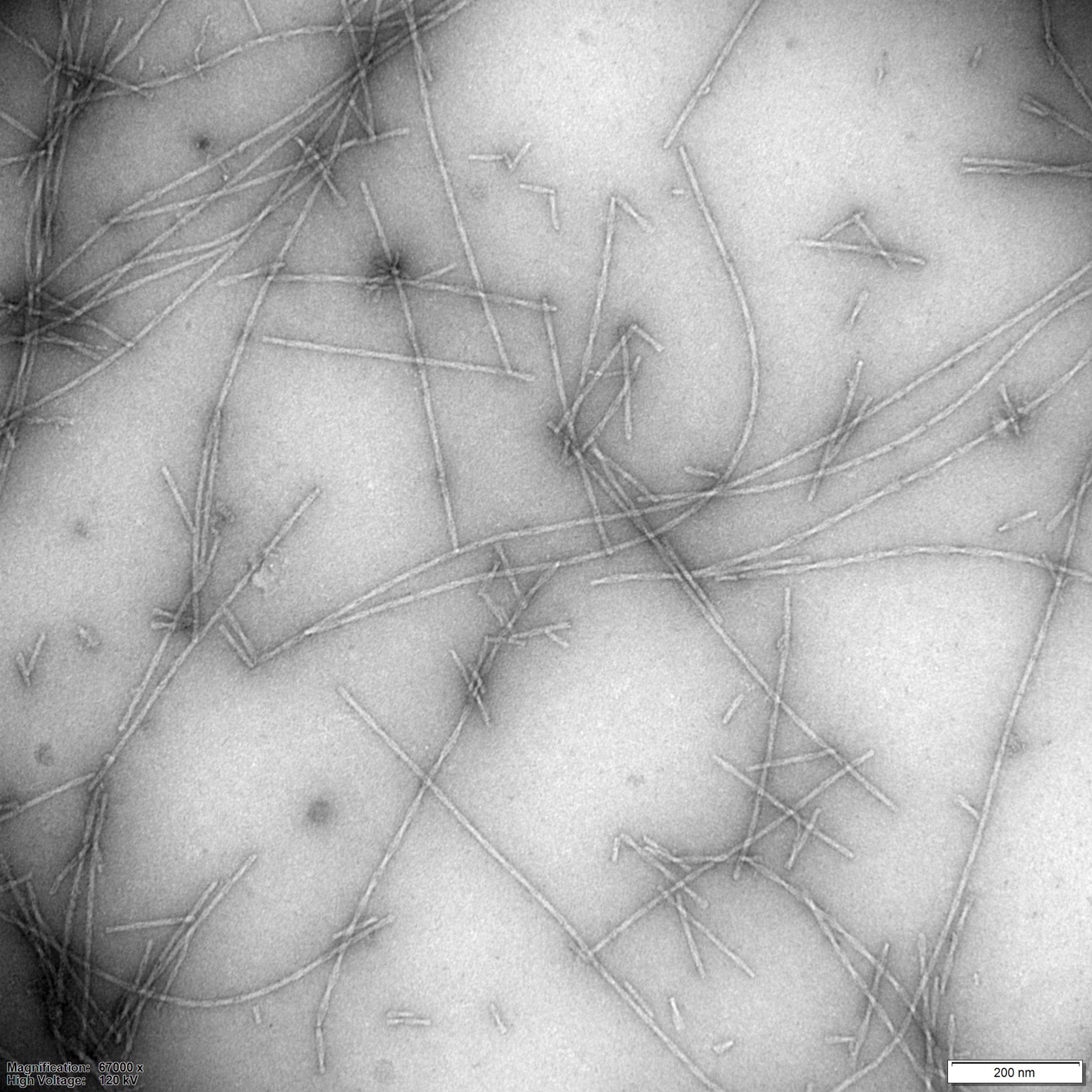


**Figure S4**. Full-scale TEM image of the C-36 fibrils illustrated in figure 1.


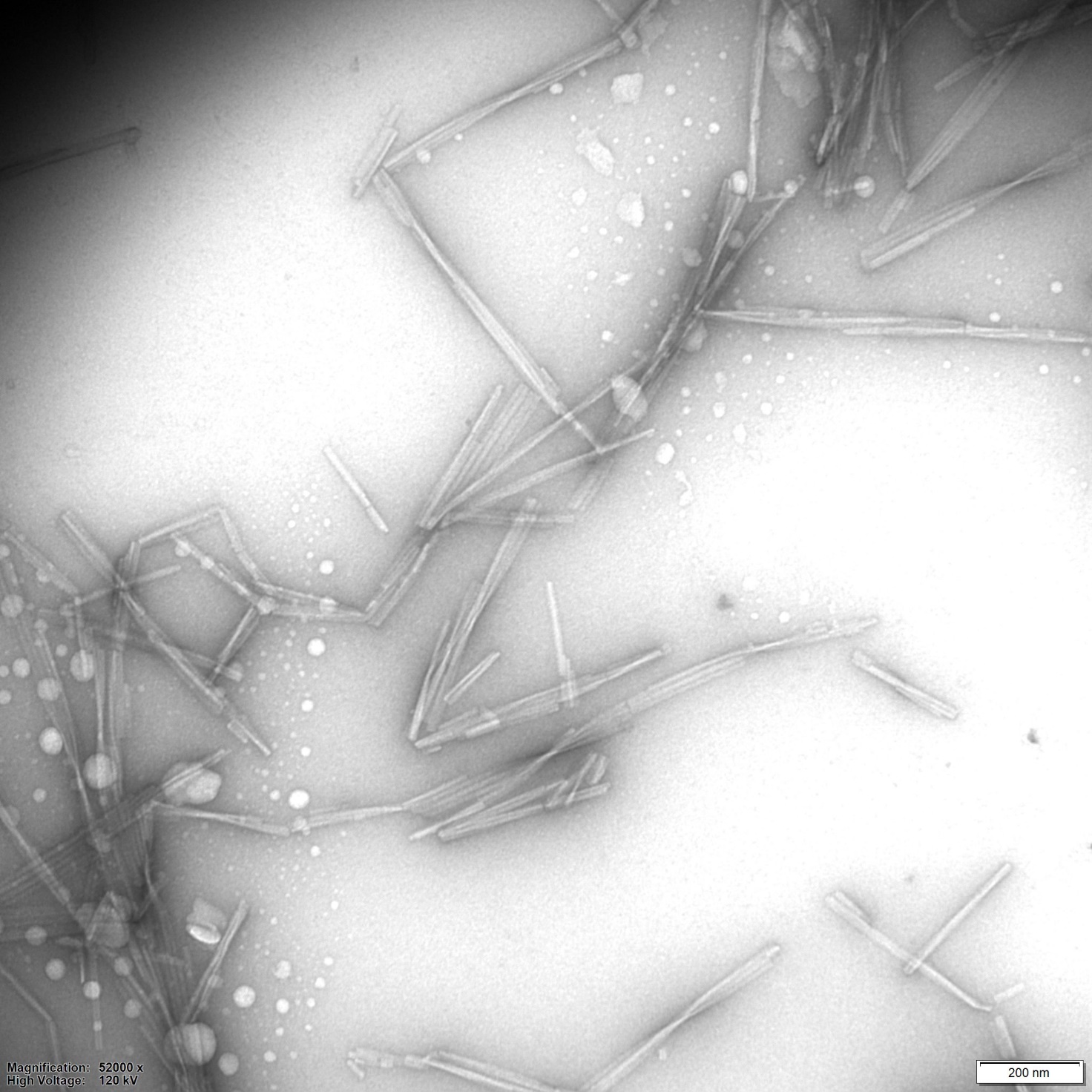


**Figure S5**. Full-scale TEM image of the a-Synuclein fibrils illustrated in figure 1.


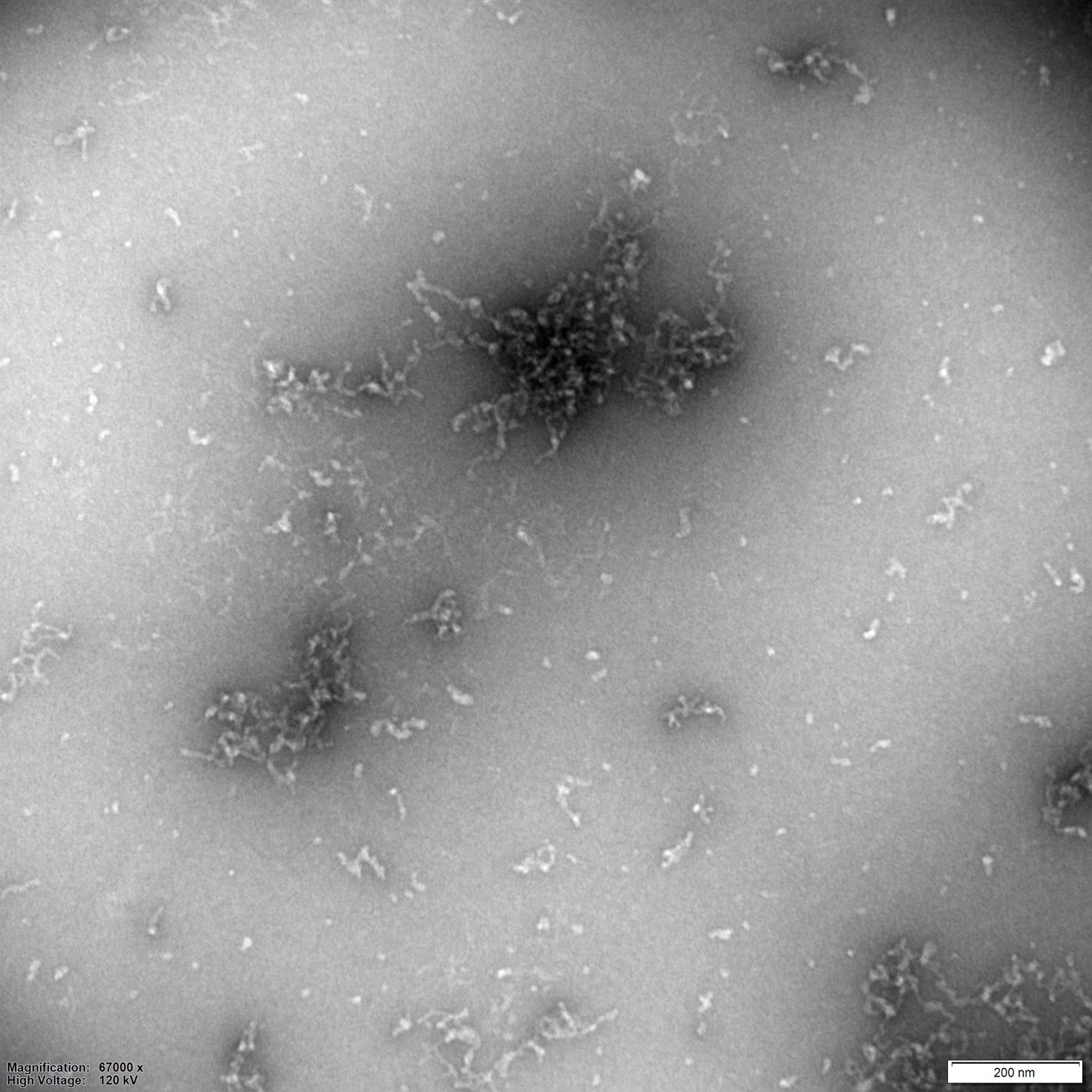


**Figure S6.** Full-scale TEM image of the FAS4 AD aggregates illustrated in figure 1.

**Figure S7**. Overlay of FTIR spectra of all fibril and aggregate samples. Each analysis was normalized to the peak intensity.

**Figure S8**. ANS fluorescence intensity (Ex 355 nm, Em 485 nm) as a function of the ANS concentration co-incubated with 2.5 μg of aggregate material.


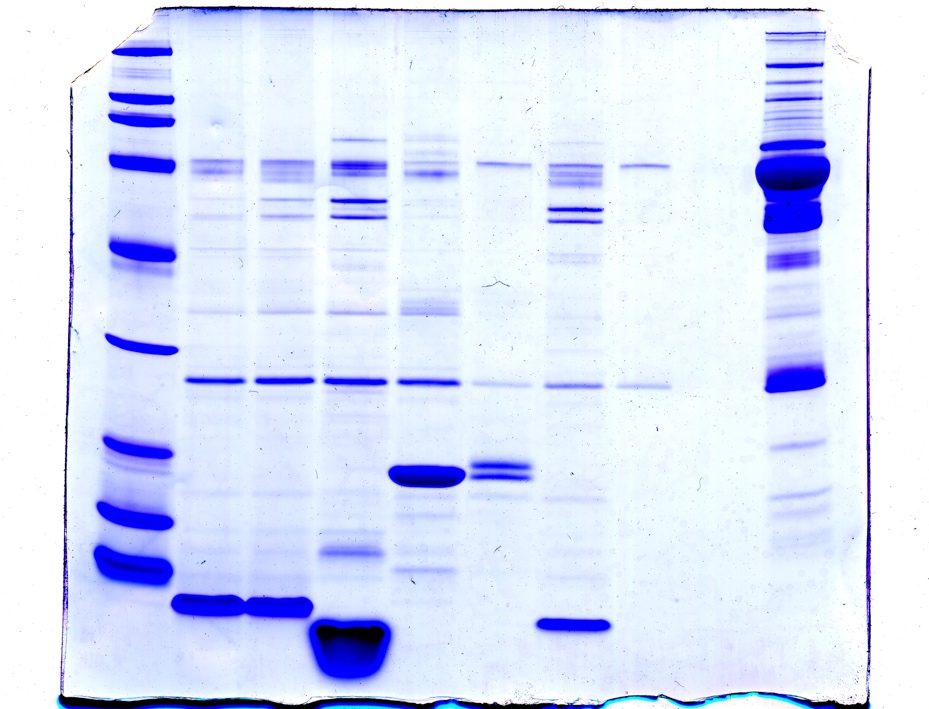


**Figure S9**. Entire SDS-PAGE gel analysed in figure 3. Fibril material (10 μg) was incubated with 500 μg plasma proteins prior to the analysis.


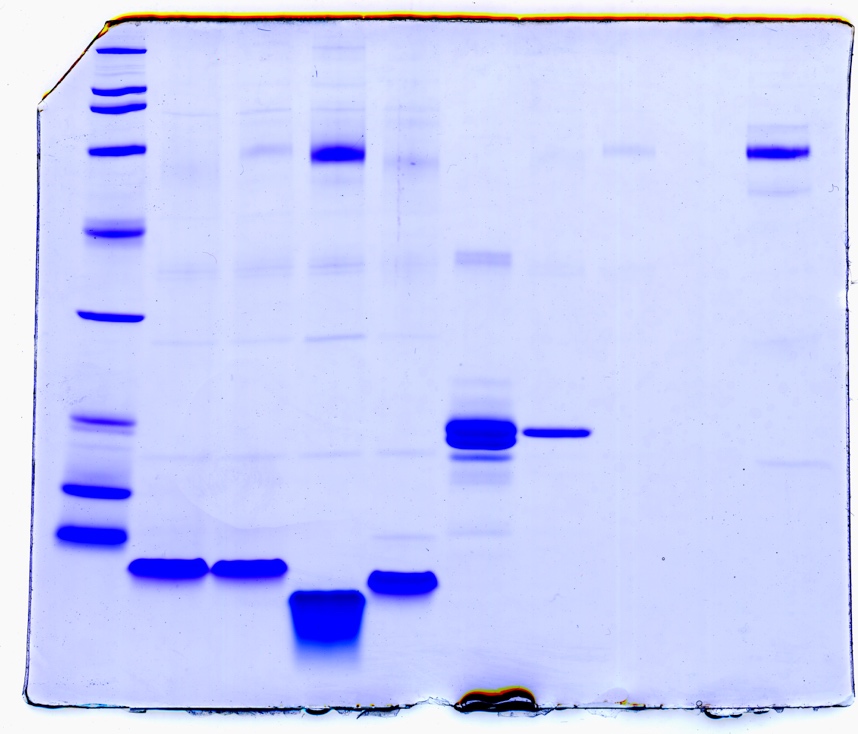


**Figure S10**. Entire SDS-PAGE gel analysed in figure 3. Fibril material (10 μg) was incubated with 40 μg CSF proteins prior to the analysis.


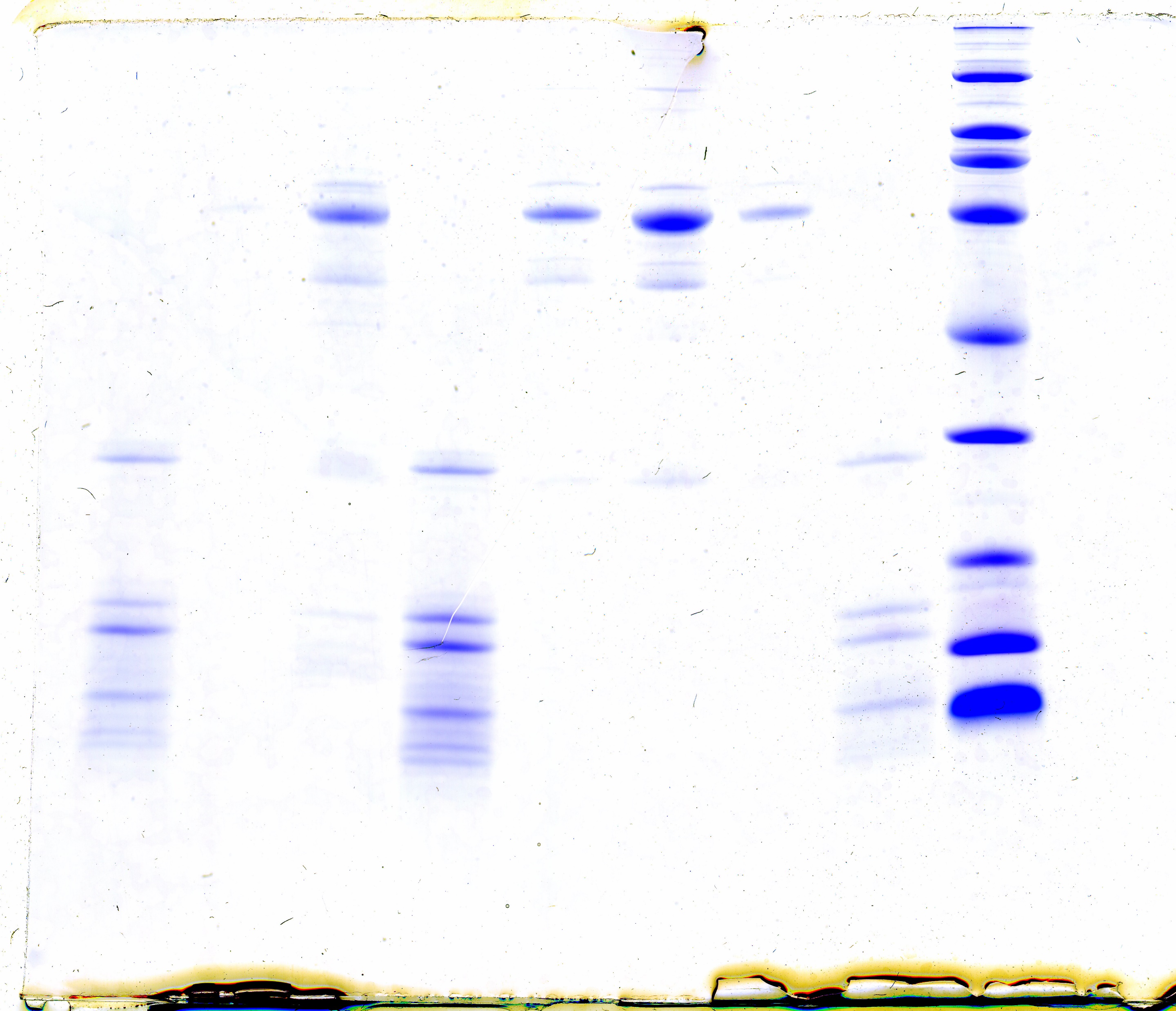


**Figure S11**. Entire SDS-PAGE gel analysed in figure 3. From left to right the lanes include: 1: Chymotrypsinogen (10 μg) incubated with 40 μg CSF proteins, 2: CSF aggregation control, 3: CSF solution, 4: Chymotrypsinogen (10 μg) incubated with 500 μg plasma proteins, 5-7: Plasma aggregation control at increasing concentrations, 8: Molecular weight marker.


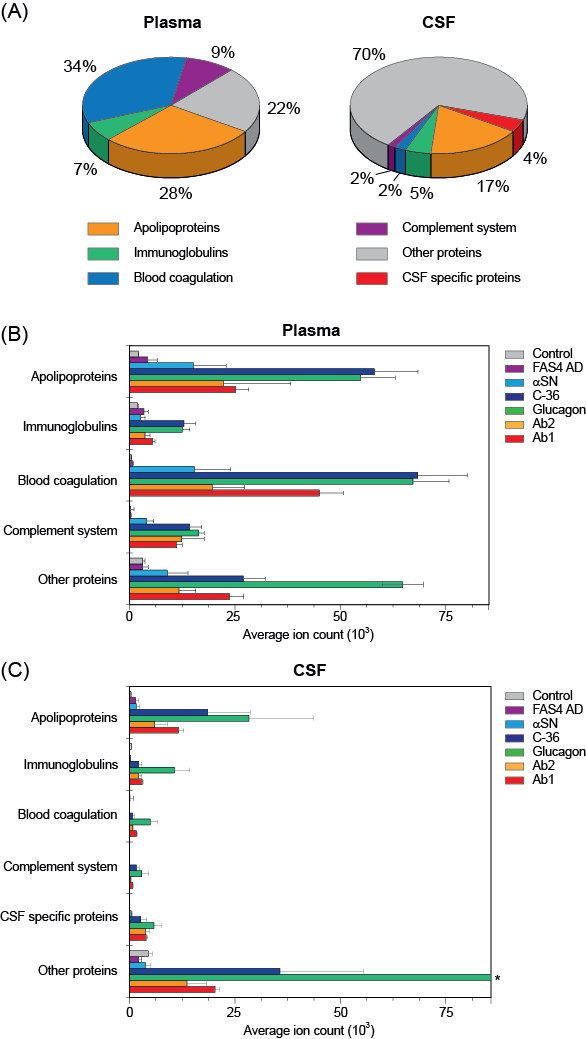


**Figure S12.** Quantitative analysis of the Amyloid Interactome. *A*, Composition of proteins in the amyloid interactome combined for all the fibril systems in plasma and CSF. *B* and *C*, Absolute ion count for each protein group divided into fibril systems for plasma and CSF, respectively. * The ion count for Other proteins (Serum albumin) in the Glucagon samples incubated with CSF is 186 ± 96 (×1000). The data represents three sample replicas, all analysed three times by MS. The error bars show the standard deviations between all nine MS/MS experiments.
